# The Quantum Friction and Optimal Finite-Time Performance of the Quantum Otto Cycle

**DOI:** 10.3390/e22091060

**Published:** 2020-09-22

**Authors:** Andrea R. Insinga

**Affiliations:** DTU Energy Conversion and Storage, Technical University of Denmark Anker Engelundsvej, Building 301, 2800 Kgs. Lyngby, Denmark; aroin@dtu.dk

**Keywords:** quantum thermodynamics, maximum power, shortcut to adiabaticity, quantum friction, Otto cycle, quantum engine, quantum refrigerator

## Abstract

In this work we considered the quantum Otto cycle within an optimization framework. The goal was maximizing the power for a heat engine or maximizing the cooling power for a refrigerator. In the field of finite-time quantum thermodynamics it is common to consider frictionless trajectories since these have been shown to maximize the work extraction during the adiabatic processes. Furthermore, for frictionless cycles, the energy of the system decouples from the other degrees of freedom, thereby simplifying the mathematical treatment. Instead, we considered general limit cycles and we used analytical techniques to compute the derivative of the work production over the whole cycle with respect to the time allocated for each of the adiabatic processes. By doing so, we were able to directly show that the frictionless cycle maximizes the work production, implying that the optimal power production must necessarily allow for some friction generation so that the duration of the cycle is reduced.

## 1. Introduction

Quantum models of heat engines and refrigerators have been investigated extensively, especially because of the relevance of these models to the problem of cooling at extremely low temperatures, i.e., near absolute zero. The most well-studied case is the quantum analog of the Otto cycle [1,2,3,4] for which heat-exchange and work-exchange take place in different steps of the thermodynamic cycle, although the Carnot cycle has been investigated as well [5,6].

We consider the typical optimization perspective assumed in the field of finite-time thermodynamics: maximization of the average power extracted from a heat-engine [7,8,9] or the average cooling power provided by a refrigerator [3,10].

In the seminal works by Berry [11] and by Rezek et al. [12] it has been shown that finite-time cycles can be constructed such that quantum friction is entirely suppressed. This result is surprising since intuition would suggest that frictionless operation could only be achieved in the quasi-static regime, i.e., cycles of infinite duration. The attainability of frictionless finite-time quantum processes has been experimentally confirmed by Deng et al. [13].

Salamon et al. [14] showed that these frictionless adiabatic trajectories maximize the work exchanged with the system with respect to the compression/expansion time-law. This is due to the suppression of quantum friction which would otherwise cause part of the exchanged energy to be spent in increasing the coherence of the system. Similarly to other relevant studies, such as those by Abah et al. [15,16], frictionless trajectories have been shown to be the optimal finite-time processes that connect two different thermal states while guaranteeing maximal work extraction, i.e., equal to that obtained in the quasi-static limit.

These analyses are often very insightful [1,17,18], due to the fact that the resulting cycles are mathematically simpler to investigate, thereby admitting analytical computation of, e.g., power and efficiency. Frequently, cycles for which the power is optimized with the constraint of frictionless adiabats are referred to as maximum-power cycles. The argument behind this statement is that the maximum realizable work can be obtained in finite time, but there is a lower bound to the minimum time required to achieve this effect. This observation would appear to suggest that the minimum-time frictionless cycle has to correspond to maximum power.

However, as we argue in the present work, the frictionless cycles are not truly maximizing the power with respect to the time allocated for the cycle. In fact, by relaxing the requirement of frictionless adiabats it is possible to reduce the time allocation for the adiabatic processes, thereby improving the power extraction, although the work extraction per cycle is slightly reduced as well. Evidence for this argument has already been presented by employing numerical methods [19]. Moreover, the experimental realization of a quantum engine demonstrated by Peterson et al. [2] also revealed that maximal power production is obtained by a time allocation that is shorter than that of a maximal-work cycle.

Here we consider general limit-cycle trajectories as functions of the time allocation for the adiabatic processes. By employing analytical calculations, we explicitly show for the first time that the special frictionless cycles provide maximum work extraction over the whole cycle. Since the average power is the work divided by the total duration of the cycle, maximum-work cycles cannot simultaneously be maximum-power cycles. It is convenient to allow for a small amount of friction production, which slightly reduces the work extraction, in order to reduce the total duration of the cycle and maximize the average power.

The case of a quantum heat engine based on harmonic oscillators is used here as a prototypical system and is analyzed in detail. Subsequently, we consider generalization of the results to other relevant cases. In particular, we also consider the case of maximum cooling power for a quantum refrigerator based on harmonic oscillators. We also consider quantum heat engines and refrigerators with different working fluids, namely, an ensemble of spin systems. All these other cases are shown to be analogous to the harmonic heat engine in that frictionless cycles are not providing maximal power with respect to the time allocation.

## 2. Framework

### 2.1. Notation and Units

In this paper, the calligraphic typeface (e.g., W) is used for scalar quantities, underlined uppercase letters (e.g., $\underline{X}$) are used for column vectors, underlined lowercase letters (e.g., $\underline{w}$) are used for row vectors and bold letters (e.g., U) are used for matrices.

Superscripts correspond to the row indexes and subscripts to the column indexes. For example ${\left(AB\right)}_{3}^{2}$ indicates the entry on the second row and third column of the product between the matrix A and the matrix B. Operators are denoted with the $\hat{\;}$ symbol, as in $\hat{X}$. Super-operators are linear operators having operators as input and output arguments. The letter L in calligraphic font is used for super-operators. For example, $L_{H}\left(\hat{X}\right)$ denotes the super-operator $L_{H}$ applied to the operator $\hat{X}$.

Moreover, in this work we assume that the mass *m* of the oscillators, the Boltzmann constant *k* and the reduced Planck constant *ℏ* are all equal to 1.

### 2.2. Governing Equations

We briefly review here the mathematical formalism discussed in reference [12,19]. The working fluid of the engine is an ensemble of identical quantum harmonic oscillators. The corresponding Hamiltonian operator $\hat{H}$ is parameterized by the angular frequency ω and the mass *m* of each oscillator:

(1) $$\hat{H}\left(t\right)=\frac{1}{2m}{\hat{P}}^{2}+\frac{1}{2}m{\left(\omega \left(t\right)\right)}^{2}\;{\hat{Q}}^{2}$$

where $\hat{Q}$ and $\hat{P}$ are the position operator and momentum operator, respectively. In the Heisenberg formalism, the time-evolution of a Hermitian operator $\hat{X}$ is described by the following equation of motion:

(2) $$\frac{d}{dt}\hat{X}\left(t\right)=L_{H}^{*}\left(\hat{X}\left(t\right)\right)+L_{D}^{*}\left(\hat{X}\left(t\right)\right)+\frac{\partial }{\partial t}\hat{X}\left(t\right),$$

where $L_{H}^{*}$ and $L_{D}^{*}$ denote the unitary and non-unitary Liouville super-operators, respectively [1]. The unitary super-operator $L_{H}^{*}$ describes the evolution of a closed system, whose Hamiltonian may be explicitly time-dependent. The super-operator $L_{H}^{*}$ is given by:

(3) $$L_{H}^{*}\left(\hat{X}\left(t\right)\right)=\frac{i}{\hbar }\left[\hat{H},\hat{X}\right]$$

For an open system [20,21], i.e., coupled to a thermal reservoir, it is necessary to include the additional non-unitary super-operator $L_{D}^{*}$. For the harmonic oscillator, the non-unitary super-operator is given by:

(4) $$L_{D}^{*}\left(\hat{X}\left(t\right)\right)=k_{↓}\left({\hat{a}}^{†}\hat{X}\hat{a}-\frac{1}{2}\left\{{\hat{a}}^{†}\hat{a},\hat{X}\right\}\right)+k_{↑}\left(\hat{a}\hat{X}{\hat{a}}^{†}-\frac{1}{2}\left\{\hat{a}{\hat{a}}^{†},\hat{X}\right\}\right).$$

where $k_{↓}$ and $k_{↑}$ are the transition rates, $\hat{a}=2^{-1/2}\left({\left(m\omega /\hbar \right)}^{1/2}\hat{Q}+i{\left(1/(m\omega \hbar )\right)}^{1/2}\hat{P}\right)$ is the annihilation operator, its Hermitian conjugate ${\hat{a}}^{†}$ is the creation operator and the curly brackets denote the anti-commutator between two operators.

The form assumed by Equation (2) depends on which Lie algebra of Hermitian operators has been chosen [8,22]. In this work, we consider the set $\left\{\hat{H},\hat{L},\hat{C},\hat{1}\right\}$, where $\hat{L}$ denotes the Lagrangian operator, $\hat{C}=\left(\omega /2\right)\left(\hat{Q}\hat{P}+\hat{P}\hat{Q}\right)$ denotes the position-momentum correlation operator and $\hat{1}$ denotes the identity operator. This set of operators, together with the Lie bracket consisting of taking the commutator between two operators, forms a Lie algebra. It can be shown that the algebra is closed with respect to the time-evolution described by Equation (2). Therefore, denoting by $\underline{X}$ the vector of expectation values ${\left(\langle \hat{H}\rangle ,\langle \hat{L}\rangle ,\langle \hat{C}\rangle ,\langle \hat{1}\rangle \right)}^{T}$, the linear equation of motion (2) is expressed as:

(5) $$\frac{d}{dt}\underline{X}=A\underline{X}$$

The matrix A is obtained by plugging each of the operators $\left\{\hat{H},\hat{L},\hat{C},\hat{1}\right\}$ in Equation (2) and applying the commutation rules derived from the canonical commutation relation $[\hat{Q},\hat{P}]=i\hbar$.

During the adiabatic processes, i.e., when the ensemble of oscillators is decoupled from the thermal reservoir, the matrix A is given by:

(6) $$A=\omega \left(t\right)\left(\begin{array}{cccc} \mu & -\mu & 0 & 0\\ -\mu & \mu & -2 & 0\\ 0 & 2 & \mu & 0\\ 0 & 0 & 0 & 0 \end{array}\right)$$

where μ denotes the dimensionless non-adiabatic parameter; i.e., $\mu =\dot{\omega }/\omega^{2}$. In this work the parameter μ is assumed to be constant during each of the adiabatic processes, leading to the following time-evolution law [1]:

(7) $$\omega \left(t\right)=\frac{\omega (0)}{1-\mu \omega (0)t}$$

During the isochoric processes the frequency ω is constant, while heat is flowing between the ensemble of oscillators and one of the thermal reservoirs. The matrix A for these steps of the cycle is given by:

(8) $$A=\left(\begin{array}{cccc} -\Gamma & 0 & 0 & +\Gamma H_{\mathrm{eq}}\\ 0 & -\Gamma & -2\omega & 0\\ 0 & +2\omega & -\Gamma & 0\\ 0 & 0 & 0 & 0 \end{array}\right)$$

where $\Gamma =k_{↓}-k_{↑}$ denotes the heat conductance and $H_{\mathrm{eq}}$ denotes the thermal equilibrium energy, which is a function of the temperature *T* of the heat reservoir. By inspecting Equation (8), we notice that the dynamic evolution of $\langle \hat{L}\rangle$ and $\langle \hat{C}\rangle$, which during the isochoric processes is decoupled from the evolution of $\langle \hat{H}\rangle$ and $\langle \hat{1}\rangle$, corresponds to a rotation with frequency 2ω accompanied by a decrease of the rotation amplitude. In the long-time limit the amplitude of the rotations approaches zero, thereby leading to a zero-coherence state for which $\langle \hat{L}\rangle =\langle \hat{C}\rangle =0$, and $\langle \hat{H}\rangle =H_{\mathrm{eq}}$.

The formal solution of Equation (5) is given by the following time-evolution equation:

(9) $$\underline{X}\left(t\right)=U\left(t\right)\underline{X}\left(0\right)$$

where U is called time-evolution matrix, and $\underline{X}\left(0\right)$ is the vector defining the initial state at t=0.

We consider the quantum Otto cycle, which consists of four processes. It is customary to employ the same terminology used for the classical Otto cycle. The rationale behind this analogy is that when the frequency ω is larger, the oscillators composing the working fluid are more tightly confined, which corresponds to a smaller available volume. Conversely, when the frequency is smaller the oscillators are less tightly confined, corresponding to a larger volume. Therefore, the two steps for which the frequency is held constant are called hot and cold isochoric processes, depending on which of the two thermal reservoirs is contact with the working medium, i.e., the hot or cold heat reservoir, respectively. In some works the term iso-frequency is used to refer to the isochoric processes. Following the analogy with the classical Otto cycle, the step for which the frequency is decreasing is called expansion adiabat, and the step for which the frequency is increasing is called compression adiabat.

Each of the four processes of the cycle is assigned a time-evolution matrix. In particular, we denote by $U_{H}$, $U_{HC}$, $U_{C}$ and $U_{CH}$, the time-evolution matrices for the hot isochore, the expansion adiabat, the cold isochore and the compression adiabat, respectively. The closed-form expression of each of the time-evolution matrices has previously been derived [1,12]. Therefore, the time-evolution matrix for one entire cycle, denoted simply by U, is given by the ordered composition of the four individual matrices:

(10) $$U=U_{CH}U_{C}U_{HC}U_{H}$$

Analogous notation is also used for the time allocated for each of the four processes:

(11) $$\tau =\tau_{H}+\tau_{HC}+\tau_{C}+\tau_{CH}$$

The temperatures for the hot and cold thermal reservoirs are denoted by $T_{H}$ and $T_{C}$, respectively. The frequencies for the hot and cold isochoric processes are denoted by $\omega_{H}$ and $\omega_{C}$, respectively.

It is important to stress that Equation (11) must not be interpreted as a constraint. In fact, the unconstrained optimization problem considered in this paper is the optimization of the average power with respect to the four times allocated for the four processes composing the Otto cycle. In other words, the total duration τ of the cycle is not predetermined. We are interested in the behavior of the heat machine at steady state, also called limit cycle, for which the state of the system is the same at the beginning and at the end of each cycle.

### 2.3. Frictionless Cycles

Among all the possible cyclic trajectories, we consider a special class, called frictionless cycles. For the time-dependence described by Equation (7) (i.e., constant μ), these special trajectories can be obtained by suitably selecting the times allocated for the adiabatic processes, i.e., $\tau_{HC}$ and $\tau_{CH}$. The details can be found in reference [12,14]. In brief, the dynamic of sufficiently slow, i.e., μ<2, adiabatic processes describe an oscillation overlapping a slower drift of the frame of reference. If the values of $\tau_{HC}$ and $\tau_{CH}$ are selected so that an integer number of such oscillations occurs, the process maps a zero-coherence initial state, i.e., one for which $\langle \hat{L}\rangle =\langle \hat{C}\rangle =0$, into another zero-coherence final state with different energy. If this is true for both the adiabatic processes, the resulting limit-cycle will maintain the property $\langle \hat{L}\rangle =\langle \hat{C}\rangle =0$ for the whole duration of each of the isochoric processes. The condition of having an integer number of oscillations limits the allowed values of adiabat times to a countable set. These times will be denoted by $\tau_{n}^{*}$, where *n* is a positive integer.

It is worth mentioning that the strategy of selecting these special cycles is sometimes called shortcut to adiabaticity [16,23,24]. The reason is that the effect $\langle \hat{L}\rangle =\langle \hat{C}\rangle =0$ can also be obtained in the quasi-static limit: i.e., when $\tau_{H},\tau_{HC},\tau_{C},\tau_{CH}\to +\infty$. The adiabatic theorem predicts that in the quasi-static regime the occupation probability of each of the energy levels remains constant during the adiabatic processes. Consequently, the amount of energy lost to quantum friction is zero. However, it is somewhat surprising that the same effect can also be attained in finite-time by a suitable selection of the adiabat times, hence the term shortcut to adiabaticity.

Besides properly selecting $\tau_{HC}$ and $\tau_{CH}$ with ω time-dependence characterized by constant μ, frictionless adiabat processes can also be obtained by considering different protocols ω(t). Most notably, a bang–bang type solution [14] leads to frictionless cycles with the additional advantage of minimizing the time allocated for the adiabatic process. The minimum time bang–bang ω(t) evolution is composed of five steps: an initial sudden transition to the final frequency; a wait of duration $\tau_{1}$; a sudden transition back to the initial frequency; a wait of duration $\tau_{2}$; and finally, one more sudden transition to the final frequency. The waiting periods $\tau_{1}$ and $\tau_{2}$ are determined by the initial and final frequencies. The total duration of the adiabatic process is thus constrained to $\tau_{1}+\tau_{2}$.

**Figure 1:**
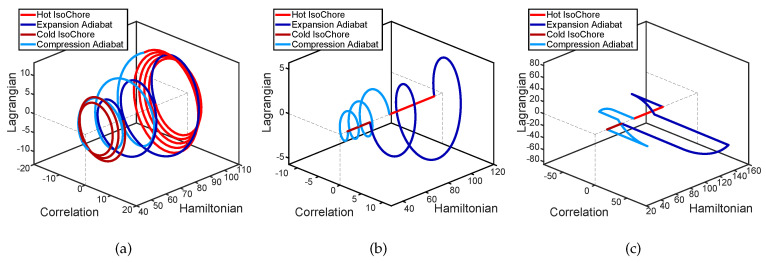
Three limit cycles corresponding to different adiabatic processes. For the cycles shown in (**a**,**b**) the ω(t) time dependence is characterized by constant μ. For the case of (**b**) the adiabat time allocations $\tau_{HC}$ and $\tau_{CH}$ are chosen among the frictionless set $\left\{\tau_{n}^{*}\right\}$, while for the case of (**a**) they are not. (**c**) A frictionless bang–bang cycle.

Figure 1 illustrates three different cycles by showing the time-evolution of the vector ${\left(\langle \hat{H}\rangle ,\langle \hat{L}\rangle ,\langle \hat{C}\rangle \right)}^{T}$, i.e., the first three components of the vector $\underline{X}$ introduced in Section 2.2. The cycle shown in Figure 1a corresponds to constant μ adiabatic processes for which the allocated times are not selected among the frictionless set $\left\{\tau_{n}^{*}\right\}$. Figure 1b shows a constant μ frictionless cycle and Figure 1c shows a minimum time bang–bang frictionless cycle. As can be noticed, for frictionless cycles the condition $\langle \hat{L}\rangle =\langle \hat{C}\rangle =0$ is satisfied for the whole duration of the isochoric processes.

The search for frictionless solutions can also be seen from a different perspective, closely related to that of searching for the optimal control ω(t). In this framework, an additional driving Hamiltonian is added to the original Hamiltonian [25] to counteract the non-adiabatic effects that are normally present for a finite-time process. By doing so, the transitions between different eigenstates of the original Hamiltonian can be entirely suppressed. For this reason, this strategy is often called transitionless quantum driving [11,26].

## 3. Analytical Results

In this work we consider the following optimization problem: maximize the average power extraction of the engine with respect to the four variables $\tau_{H}$, $\tau_{HC}$, $\tau_{C}$ and $\tau_{CH}$, i.e., the time allocations for each of the four processes.

As mentioned in the introduction, we will show that the trajectories leading to maximum average power are not frictionless cycles. First, we will show in Section 3.1 that frictionless cycles are relative maxima of the total work extracted during a cycle with respect to the times $\tau_{HC}$ and $\tau_{CH}$ allocated during the adiabatic processes. Then in Section 3.2 we show that in order to maximize the average power it is convenient to reduce the durations of the adiabat times, thereby allowing for some quantum friction to be generated.

In Section 3.3 we show an analogous result for a quantum refrigerator, where the optimization objective is the maximization of the average cooling power. Finally, in Section 3.4 we argue why identical results are also applicable to heat engines and refrigeration having as working fluid an ensemble of spin systems.

### 3.1. Maximum Work

We want to show that for a constant μ frictionless trajectory the total work $W_{\mathrm{tot}}$ extracted from the system during one cycle is locally optimal with respect to the adiabat times:

(12) $${\left(\frac{\partial W_{\mathrm{tot}}}{\partial \tau_{HC}}\right)}_{\begin{array}{c} \tau_{HC}=\tau_{n}^{*}\\ \tau_{CH}=\tau_{m}^{*} \end{array}}={\left(\frac{\partial W_{\mathrm{tot}}}{\partial \tau_{CH}}\right)}_{\begin{array}{c} \tau_{HC}=\tau_{n}^{*}\\ \tau_{CH}=\tau_{m}^{*} \end{array}}=0\;\forall n,m,\tau_{H},\tau_{C}$$

We start from the compression adiabat by showing that the derivative of $W_{\mathrm{tot}}$ with respect to $\tau_{CH}$ is zero when the trajectory is frictionless. We will then argue that the derivation is completely analogous for the expansion adiabat and $\tau_{HC}$.

The amount of heat $Q_{H}$ extracted during the hot isochore is a linear function of the initial state vector ${\underline{X}}_{\infty }$ of the limit cycle, and can thus be expressed as the scalar product between a row vector ${\underline{q}}_{H}$ and ${\underline{X}}_{\infty }$. The row vector ${\underline{q}}_{H}$ does not depend on the initial state of the system, but only on the process that the system undergoes during the hot isochoirc step. When ${\underline{q}}_{H}$ is applied to the initial state vector, the result is the amount of heat extracted during the hot isochore:

(13) $$Q_{H}={\underline{q}}_{H}{\underline{X}}_{\infty }=\sum_{k}{\left(U_{H}\right)}_{k}^{1}{\left({\underline{X}}_{\infty }\right)}^{k}-{\left({\underline{X}}_{\infty }\right)}^{1}$$

Denoting by 1 the identity matrix, the row vector ${\underline{q}}_{H}$ is defined as:

(14) $${\left({\underline{q}}_{H}\right)}_{k}={\left(U_{H}-1\right)}_{k}^{1}$$

Similarly, the amount of heat $Q_{C}$ extracted during the cold isochore is obtained from the state vector $\left(U_{HC}U_{H}{\underline{X}}_{\infty }\right)$ at the beginning of the cold isochore:

(15) $$Q_{C}={\underline{q}}_{C}{\underline{X}}_{\infty }=\sum_{k}{\left(U_{C}\right)}_{k}^{1}{\left(U_{HC}U_{H}{\underline{X}}_{\infty }\right)}^{k}-{\left(U_{HC}U_{H}{\underline{X}}_{\infty }\right)}^{1}$$

The row vector ${\underline{q}}_{C}$ is defined as:

(16) $${\left({\underline{q}}_{C}\right)}_{k}={\left(U_{C}U_{HC}U_{H}-U_{HC}U\right)}_{k}^{1}$$

At steady state the total work $W_{\mathrm{tot}}$ extracted from the system is equal to the total heat flowing into the system, and is thus given by the sum of the two contributions:

(17) $$W_{\mathrm{tot}}={\underline{w}}_{\mathrm{tot}}{\underline{X}}_{\infty }={\underline{q}}_{H}{\underline{X}}_{\infty }+{\underline{q}}_{C}{\underline{X}}_{\infty }$$

We can calculate the derivative of the work with respect to $\tau_{CH}$ as:

(18) $$\partial_{\tau_{CH}}W_{\mathrm{tot}}=\left(\partial_{\tau_{CH}}{\underline{w}}_{\mathrm{tot}}\right){\underline{X}}_{\infty }+{\underline{w}}_{\mathrm{tot}}\left(\partial_{\tau_{CH}}{\underline{X}}_{\infty }\right)$$

As we can see from Equations (14) and (16), the total work vector ${\underline{w}}_{\mathrm{tot}}$ is independent of $U_{CH}$, and its derivative with respect to $\tau_{CH}$ is thus zero. The total work $W_{\mathrm{tot}}$ depends on $\tau_{CH}$ only through the limit cycle initial vector ${\underline{X}}_{\infty }$. In order for the work to be a stationary point with respect to $\tau_{CH}$, the second term on the right-hand side of Equation (18) must be zero:

(19) $$\partial_{\tau_{CH}}W_{\mathrm{tot}}={\underline{w}}_{\mathrm{tot}}\left(\partial_{\tau_{CH}}{\underline{X}}_{\infty }\right)=0$$

For now we only need to assume that $\tau_{HC}=\tau_{n}^{*}$. It can be shown that the second and third components of ${\underline{w}}_{\mathrm{tot}}$ are zero for such a trajectory; i.e., the work only depends on $\langle \hat{H}\rangle$ and $\langle \hat{1}\rangle$. Denoting by ★ the non-zero matrix entries, we have:

(20) $$\tau_{HC}=\tau_{n}^{*}\;\Rightarrow \;{\underline{w}}_{\mathrm{tot}}=\left(\begin{array}{cccc} ★ & 0 & 0 & ★ \end{array}\right)$$

Intuitively, it is not surprising that for a frictionless trajectory the work extraction does not depend on $\langle \hat{L}\rangle$ or $\langle \hat{C}\rangle$. In fact, the evolution during the icochoric processes decouples $\hat{H}$ from $\hat{L}$ and $\hat{C}$, and the time $\tau_{HC}$ allocated for the expansion adiabat is picked in such a way that an integer number of oscillations occurs and the state of the system returns to the same accumulated phase (i.e., iΩθ=nπ), with $\hat{H}$ and $\hat{L}$ rescaled by a factor $\omega_{C}/\omega_{H}$. During the first three steps of the cycle the Hamiltonian, which determine the heat exchange, evolution is thus completely decoupled from $\hat{L}$ and $\hat{C}$, and this explains why the work, when expressed as a functionn of the state of the system at the beginning of the cycle, does not depend on $\langle \hat{L}\rangle$ or $\langle \hat{C}\rangle$.

The fourth component of $\partial_{\tau_{CH}}{\underline{X}}_{\infty }$ is clearly zero since it corresponds to the expectation value $\langle \hat{1}\rangle$ which is always equal to 1. Therefore, we only need to show that its first component is also zero. It is convenient to start from the equation expressing the invariance, with respect to the whole cycle evolution matrix U, of the limit cycle’s initial state ${\underline{X}}_{\infty }$:

(21) $$U{\underline{X}}_{\infty }={\underline{X}}_{\infty }$$

By taking the derivative with respect to $\tau_{CH}$ on both sides we get:

(22) $$\partial_{\tau_{CH}}\left(U{\underline{X}}_{\infty }\right)=\left(\partial_{\tau_{CH}}U\right){\underline{X}}_{\infty }+U\left(\partial_{\tau_{CH}}{\underline{X}}_{\infty }\right)=\left(\partial_{\tau_{CH}}{\underline{X}}_{\infty }\right)$$

Reordering, we get:

(23) $$\left(U-1\right)\left(\partial_{\tau_{CH}}{\underline{X}}_{\infty }\right)=-\left(\partial_{\tau_{CH}}U\right){\underline{X}}_{\infty }$$

The $\tau_{CH}$ derivative of ${\underline{X}}_{\infty }$ can thus be obtained by solving the linear system of equations expressed by Equation (23). The derivative $\left(\partial_{\tau_{CH}}U\right)$ is obtained from $\left(\partial_{\tau_{CH}}U_{CH}\right)$:

(24) $$\left(\partial_{\tau_{CH}}U\right)=\left(\partial_{\tau_{CH}}U_{CH}\right)U_{C}U_{HC}U_{H}$$

All the quantities appearing in Equation (23) can be easily evaluated for $\tau_{CH}=\tau_{m}^{*}$ and $\tau_{HC}=\tau_{n}^{*}$, before solving the linear system for $\partial_{\tau_{CH}}{\underline{X}}_{\infty }$. In fact, the operation of replacing the values of $\tau_{CH}$ and $\tau_{HC}$ and that of solving the system are interchangeable, but the calculation is easier if the substitution is performed before solving the system. In frictionless conditions we have the following matrix structure:

(25) $$U=\left(\begin{array}{cccc} ★ & 0 & 0 & ★\\ 0 & ★ & ★ & 0\\ 0 & ★ & ★ & 0\\ 0 & 0 & 0 & ★ \end{array}\right)\;;\;\left(\partial_{\tau_{CH}}U\right)=\left(\begin{array}{cccc} 0 & ★ & ★ & 0\\ ★ & ★ & ★ & ★\\ 0 & ★ & ★ & 0\\ 0 & 0 & 0 & 0 \end{array}\right)\;;\;{\underline{X}}_{\infty }=\left(\begin{array}{c} ★\\ 0\\ 0\\ 1 \end{array}\right)$$

The second and third components of ${\underline{X}}_{\infty }$ are zero, and also the first and fourth components of the first row of $\partial_{\tau_{CH}}U$ are zero. This implies that the first component of the vector $-\left(\partial_{\tau_{CH}}U\right){\underline{X}}_{\infty }$ on the right-hand side of Equation (23) is zero:

(26) $$-\left(\partial_{\tau_{CH}}U\right){\underline{X}}_{\infty }={\left(\begin{array}{cccc} 0 & ★ & 0 & 0 \end{array}\right)}^{T}$$

Since the matrix (U−1) decouples the first and fourth components, (i.e., $\hat{H}$ and $\hat{1}$), from the second and third components, (i.e., $\hat{L}$ and $\hat{C}$), solving the linear system shows that indeed the first component of $\partial_{\tau_{CH}}{\underline{X}}_{\infty }$ is zero:

(27) $$\partial_{\tau_{CH}}{\underline{X}}_{\infty }={\left(\begin{array}{cccc} 0 & ★ & ★ & 0 \end{array}\right)}^{T}$$

Plugging Equations (20) and (27) into Equation (19) shows that:

(28) $${\left(\frac{\partial W_{\mathrm{tot}}}{\partial \tau_{CH}}\right)}_{\begin{array}{c} \tau_{HC}=\tau_{n}^{*}\\ \tau_{CH}=\tau_{m}^{*} \end{array}}=0\;\forall n,m,\tau_{H},\tau_{C}$$

The same result can be shown for the expansion adiabat by considering a cycle in which the four steps are rearranged in such a way that the expansion adiabat is the last step (i.e., cold isochore, compression adiabat, hot isochore, expansion adiabat).

As discussed in [1], the total work for the case of frictionless trajectories can be computed analytically, and it assumes a particularly simple expression:

(29) $$W_{\mathrm{tot}}=-G_{W}\left(T_{C},\omega_{C},T_{H},\omega_{H}\right)F\left(\tau_{C},\tau_{H}\right)$$

where the function $G_{W}$ is entirely determine by the engine parameters

(30) $$G_{W}\left(T_{C},\omega_{C},T_{H},\omega_{H}\right)=\left(\frac{\omega_{H}-\omega_{C}}{e^{\omega_{H}/T_{H}}-1}-\frac{\omega_{H}-\omega_{C}}{e^{\omega_{C}/T_{C}}-1}\right)$$

and the function *F* is determined by the isochore times and the heat conductance Γ

(31) $$F\left(\tau_{C},\tau_{H}\right)=\frac{\left(e^{\Gamma \tau_{H}}-1\right)\left(e^{\Gamma \tau_{C}}-1\right)}{e^{\Gamma \tau_{C}+\Gamma \tau_{H}}-1}$$

It is important to stress that the value of $W_{\mathrm{tot}}$ for frictionless cycles remains the same regardless of the particular choice of ω(t) time dependence, i.e., constant μ or bang–bang process.

### 3.2. Maximum Power

It is now easy to show that the power cannot be optimal for frictionless trajectories. The average power ${\bar{P}}_{\mathrm{tot}}$ is defined as the work $W_{\mathrm{tot}}$ extracted during a cycle divided by the duration τ of the cycle. It is convenient to express ${\bar{P}}_{\mathrm{tot}}$ as the following product:

(32) $${\bar{P}}_{\mathrm{tot}}=W_{\mathrm{tot}}f\left(\tau_{CH}\right)$$

where the scaling function $f(\tau_{CH})$ is given by:

(33) $$f\left(\tau_{CH}\right)=\frac{1}{\tau_{H}+\tau_{HC}+\tau_{C}+\tau_{CH}}$$

Since the derivative of $f(\tau_{CH})$ is always negative, it is already apparent that the trajectories that are locally optimal for $W_{\mathrm{tot}}$ cannot be optimal for ${\bar{P}}_{\mathrm{tot}}$. From Equation (32), the derivative of ${\bar{P}}_{\mathrm{tot}}$ with respect to $\tau_{CH}$ is given by:

(34) $$\partial_{\tau_{CH}}{\bar{P}}_{\mathrm{tot}}=\left(\partial_{\tau_{CH}}W_{\mathrm{tot}}\right)\left(f\left(\tau_{CH}\right)\right)+\left(W_{\mathrm{tot}}\right)\left(\partial_{\tau_{CH}}f\left(\tau_{CH}\right)\right)$$

As shown in the previous section, the derivative $\partial_{\tau_{CH}}W_{\mathrm{tot}}$ is zero when both the conditions $\tau_{CH}=\tau_{m}^{*}$ and $\tau_{HC}=\tau_{n}^{*}$ are satisfied. Therefore, the derivative of the average power ${\bar{P}}_{\mathrm{tot}}$ with respect to $\tau_{CH}$ is given by:

(35) $$\partial_{\tau_{CH}}{\bar{P}}_{\mathrm{tot}}=\left(W_{\mathrm{tot}}\right)\left(\partial_{\tau_{CH}}f\left(\tau_{CH}\right)\right)$$

**Figure 2:**
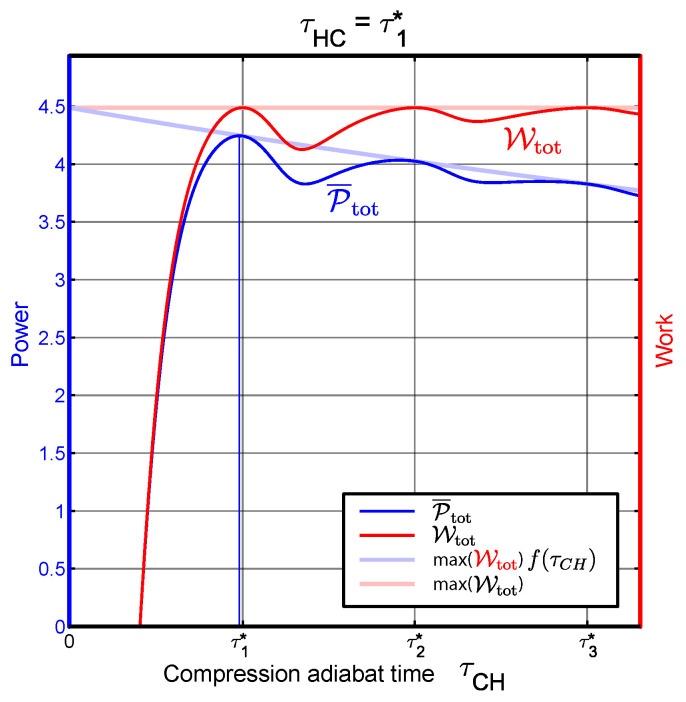
Shift of the power optimal point with respect to $\tau_{CH}$. The vertical grid lines correspond to $\tau_{CH}=\tau_{n}^{*}$ for n=1,2,3. The expansion adiabat time is $\tau_{HC}=\tau_{1}^{*}$. The isochore times are constant: $\tau_{H}=\tau_{C}=1.24$.

Since $\partial_{\tau_{CH}}f\left(\tau_{CH}\right)<0$ and $W_{\mathrm{tot}}>0$ the derivative is negative. It is thus convenient to reduce $\tau_{CH}$ from $\tau_{m}^{*}$ and allow for some friction generation in order to reduce the total cycle time and increase the average power ${\bar{P}}_{\mathrm{tot}}$. These arguments are illustrated in Figure 2. Work and average power are plotted as functions of the compression adiabat time. The expansion adiabat time is $\tau_{HC}=\tau_{1}^{*}$, thereby leading to a frictionless cycle when $\tau_{CH}\in \left\{\tau_{n}^{*}\right\}$. As can be seen from the graph, the work is maximized for these choices, always leading to the value expressed by Equation (29). On the other hand, the maximum power is obtained when $\tau_{CH}$ is slightly smaller than $\tau_{1}^{*}$. The results shown in Figure 2 correspond to the following choice of parameters: $\omega_{C}=15$, $\omega_{H}=30$, $T_{C}=100/3$, $T_{H}=125$, Γ=0.7.

So far it was implicitly assumed that around the point $\tau_{CH}=\tau_{n}^{*}$, $\tau_{HC}=\tau_{m}^{*}$ the work extraction $W_{\mathrm{tot}}$ is differentiable with respect to the time allocations $\tau_{HC}$ and $\tau_{CH}$ and that the derivative is continuous around that point. By considering the definitions [1] of the time evolution matrices $\left\{U_{H},U_{HC},U_{C},U_{CH}\right\}$, it is easy to show that they are continuously differentiable functions of all the parameters, including the time allocations. Therefore, the same property is satisfied by the row vectors $\left\{{\underline{w}}_{\mathrm{tot}},{\underline{q}}_{H},{\underline{q}}_{C}\right\}$, since these are defined from the evolution matrices (see Equations (14) and (16)). The only possible source of discontinuity is thus the limit-cycle state vector ${\underline{X}}_{\infty }$. The vector ${\underline{X}}_{\infty }$ could indeed be not continuously differentiable since it is defined as the solution of the linear system expressed by Equation (21). In other words, the calculation of ${\underline{X}}_{\infty }$ involves a matrix inversion, which can bring about a discontinuity. As discussed in reference [22], this happens in the presence of a bifurcation. However, as shown in reference [19,22], a bifurcation never occurs in a neighborhood of a frictionless cycle, thereby guaranteeing that $W_{\mathrm{tot}}$ is continuously differentiable around its maxima.

### 3.3. Harmonic Refrigerator

We argue here that the same results apply to the optimization of the cooling power of a refrigerator.

The machine will behave as an engine or a refrigerator depending on the values of the compression ratio $\omega_{H}/\omega_{C}$ and the temperature ratio $T_{H}/T_{C}$. In particular, as long as $\omega_{H}/\omega_{C}<T_{H}/T_{C}$ the machine will act as a heat engine, whereas when $\omega_{H}/\omega_{C}>T_{H}/T_{C}$ the machine behaves as a refrigerator.

The procedure of the previous section can be applied to a refrigerator by replacing the work vector ${\underline{w}}_{\mathrm{tot}}$ with the cold heat vector ${\underline{q}}_{C}$. Since the plus sign means that energy is flowing into the system from the cold heat reservoir, the objective is to maximize the cooling power (i.e., $Q_{C}$ divided by the total duration of a cycle). Using the same notation adopted in the previous section, we write:

(36) $${\bar{\dot{Q}}}_{C}=Q_{C}f\left(\tau_{CH}\right),\;\mathrm{with}\;f\left(\tau_{CH}\right)=\frac{1}{\tau_{H}+\tau_{HC}+\tau_{C}+\tau_{CH}}$$

As in the previous case ${\underline{q}}_{C}$ is independent of $U_{CH}$ and so $\partial_{\tau_{CH}}{\underline{q}}_{C}=0$. Moreover, the second and third components of ${\underline{q}}_{C}$ are still zero, because of the same arguments presented in the previous section: for a frictionless steady-state trajectory the heat transfer does not depend on the initial values of $\langle \hat{L}\rangle$ or $\langle \hat{C}\rangle$.

The limit cycle is calculated in the same way as for a heat engine. The derivative with respect to $\tau_{CH}$ of the first component of ${\underline{X}}_{\infty }$ is zero for a frictionless cycle. This property remains true regardless of the choice of parameters (i.e., for $\omega_{H}/\omega_{C}<T_{H}/T_{C}$ corresponding to a heat engine, or for $\omega_{H}/\omega_{C}>T_{H}/T_{C}$ corresponding to a refrigerator).

In conclusion, frictionless trajectories correspond to the locally optimal cold heat transfer with respect to $\tau_{CH}$ and $\tau_{HC}$, implying that they cannot also give the optimal cooling power ${\bar{\dot{Q}}}_{C}$.

### 3.4. Spin System

The same results apply also to heat machines and engines having the spin system as the working fluid. These systems have been extensively studied by Kosloff and Feldmann [27,28,29].

It is still possible to select particular values of the time allocated during the adiabatic processes in order to eliminate the correlation between the Hamiltonian $\hat{H}$ and the operators $\hat{L}$ and $\hat{C}$. In this case the evolution matrices for the adiabats become diagonal. The operators are rescaled by factor that depends on the initial and final frequencies. The first 3×3 matrix block of the evolution matrix $U_{CH}$ is proportional to the identity matrix:

(37) $${\tilde{U}}_{CH}={\left(\frac{\left(J^{2}+\omega_{H}^{2}\right)}{\left(J^{2}+\omega_{C}^{2}\right)}\right)}^{1/2}\;\tilde{1}\equiv \left(\Omega_{H}/\Omega_{C}\right)\;\tilde{1}$$

This similarity with the harmonic case already shows that for a frictionless steady-state cycle the work and heat transfer vectors are independent of the values of $\langle \hat{L}\rangle$ and $\langle \hat{C}\rangle$. Moreover, the same vectors ${\underline{w}}_{\mathrm{tot}}$ and ${\underline{q}}_{C}$ are independent of $\tau_{CH}$.

We thus only have to show that the derivative with respect to $\tau_{CH}$ of the first component (i.e., $\langle \hat{H}\rangle$) of the initial state is zero for a frictionless cycle. The same procedure employed in the previous section can be used to show that this property holds also for the spin case.

## 4. Numerical Results

While the case of frictionless trajectories can be treated by employing analytical techniques, the general case involves complicated mathematical expressions which cannot be manipulated analytically for the purpose of obtaining the maximum power. Therefore, we resort to numerical methods to compute the performance improvement with respect to the frictionless case. We denote by ${\bar{P}}_{\mathrm{tot}}\left(\tau_{H},\tau_{HC},\tau_{C},\tau_{CH}\right)$ the average power as a function of the time allocations for the four processes.

In order to reduce the computation time, we consider a simplified case as an example: We assume that the time allocated for the hot isochore is the same as the time allocated for the cold isochore, and it will be denoted by $\tau_{\mathrm{is}.}$. Furthermore, we assume that the time allocated for the compression adiabat is the same as the time allocated for the expansion adiabat, and it will be denoted by $\tau_{\mathrm{ad}.}$. We performed numerical optimization of the average power ${\bar{P}}_{\mathrm{tot}}$ as a function of these two parameters: $\tau_{\mathrm{is}.}$ and $\tau_{\mathrm{ad}.}$. Due to the analytical results presented in the previous sections, for the adiabats time we only consider the interval $\left[0,\tau_{1}^{*}\right]$.

**Figure 3:**
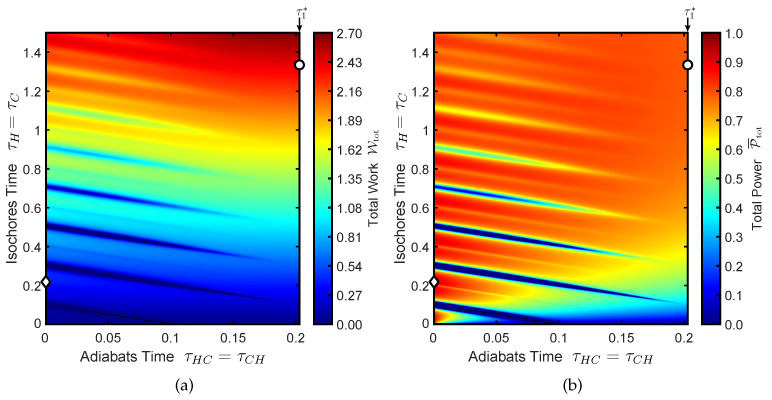
The work and power landscape as functions of adiabat and isochore times are shown in (**a**,**b**), respectively. The maximum power among frictionless cycles, ${\bar{P}}_{\mathrm{tot}}^{\mathrm{opt}(*)}$, is indicated by the white circle. This cycle is located on the right border of each graph corresponding to $\tau_{\mathrm{ad}.}=\tau_{1}^{*}$. The unconstrained maximum power cycle ${\bar{P}}_{\mathrm{tot}}^{\mathrm{opt}}$ is indicated by the white diamond. For this case the unconstrained optimum is located on the left border of each graph since it corresponds to a sudden-adiabats cycle. Although the total work is significantly less than that of the frictionless cycle, the reduction of the cycle duration is even higher, thereby resulting in higher average power.

Figure 3a shows an example of work landscape as a functionn of the two parameters and Figure 3b shows the corresponding power landscape. The optimal power among the frictionless trajectories with constant μ will be denoted by ${\bar{P}}_{\mathrm{tot}}^{\mathrm{opt}(*)}$:

(38) $${\bar{P}}_{\mathrm{tot}}^{\mathrm{opt}(*)}=\max_{\tau_{\mathrm{is}.}}\left[{\bar{P}}_{\mathrm{tot}}\left(\tau_{\mathrm{is}.},\tau_{1}^{*},\tau_{\mathrm{is}.},\tau_{1}^{*}\right)\right]$$

In Figure 3a,b the point corresponding to ${\bar{P}}_{\mathrm{tot}}^{\mathrm{opt}(*)}$ is indicated by the white circle located on the right border of the graph (i.e., $\tau_{\mathrm{ad}.}=\tau_{1}^{*}$).

The unconstrained optimal power is denoted by ${\bar{P}}_{\mathrm{tot}}^{\mathrm{opt}}$:

(39) $${\bar{P}}_{\mathrm{tot}}^{\mathrm{opt}}=\max_{\tau_{\mathrm{is}.},\tau_{\mathrm{ad}.}}\left[{\bar{P}}_{\mathrm{tot}}\left(\tau_{\mathrm{is}.},\tau_{\mathrm{ad}.},\tau_{\mathrm{is}.},\tau_{\mathrm{ad}.}\right)\right]$$

The point corresponding to ${\bar{P}}_{\mathrm{tot}}^{\mathrm{opt}}$ is indicated by the white diamond located in the left border of the graph. In fact, for this choice of engine parameters the optimal power corresponds to a sudden-adiabats cycle, despite the fact the the optimal work is given by the frictionless trajectory. It is interesting to notice that, in contrast with Figure 2, the optimal power was not found in the vicinity of a frictionless trajectory. While this result might be counter-intuitive, it highlights the fact that maximizing the power and maximizing the work are indeed very different optimization problems. The results presented in Figure 3 correspond to the following choice f engine parameters: $\omega_{C}=15$, $\omega_{H}=16$, $T_{C}=100/3$, $T_{H}=125$, Γ=0.7.

We now consider the dependence of ${\bar{P}}_{\mathrm{tot}}^{\mathrm{opt}(*)}$ and ${\bar{P}}_{\mathrm{tot}}^{\mathrm{opt}}$ on the engine parameters, i.e., the compression ratio $\omega_{H}/\omega_{C}$ and the temperature ratio $T_{H}/T_{C}$, with Γ=0.7. Since we are interested in the relative power improvement, we compute the unconstrained maximum power normalized by the maximum power among frictionless cycles with the same engine parameters, i.e.,

(40) $$\hat{P}=\frac{{\bar{P}}_{\mathrm{tot}}^{\mathrm{opt}}\left(\omega_{H}/\omega_{C},T_{H}/T_{C}\right)}{{\bar{P}}_{\mathrm{tot}}^{\mathrm{opt}(*)}\left(\omega_{H}/\omega_{C},T_{H}/T_{C}\right)}$$

**Figure 4:**
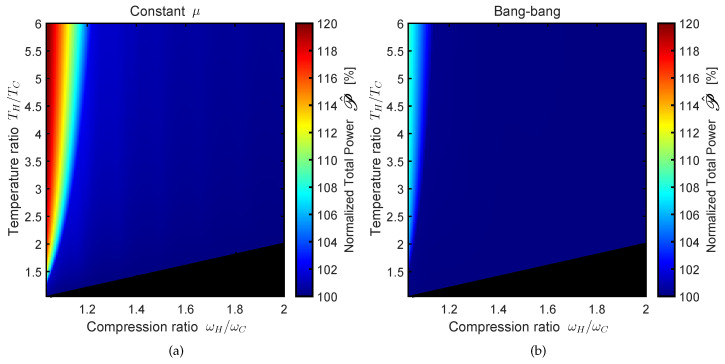
Maximum power as a function of the engine parameters $\omega_{H}/\omega_{C}$ and $T_{H}/T_{C}$. The power ${\bar{P}}_{\mathrm{tot}}^{\mathrm{opt}}$ of the unconstrained optimum is normalized by the maximum power ${\bar{P}}_{\mathrm{tot}}^{\mathrm{opt}(*)}$ among frictionless cycles. The normalized power $\hat{P}$ is expressed as a percentage. (**a**,**b**) Different ω(t) time dependence, i.e., constant μ and bang–bang, respectively.

The result is shown in Figure 4a for the ω(t) time dependence corresponding to constant μ. The black region on the bottom-right corner of the axis corresponds to the combinations of parameters for which $\left(\omega_{H}/\omega_{C}\right)>\left(T_{H}/T_{C}\right)$, i.e., leading to cooling cycles for which the working fluid behaves as a refrigerator.

As can be noticed, the maximum improvement is obtained in the limit $\omega_{H}\to \omega_{C}$ and $T_{H}\gg T_{C}$. The maximum improvement within the region shown in the figure is ≈21%, but an even greater improvement can be obtained for higher values of $T_{H}/T_{C}$.

The results shown in Figure 4b are analogous to those shown in Figure 4a except that the ω(t) time dependence is of the bang–bang type instead of constant μ. As explained in Section 2.3, the adiabat times for the frictionless cycles are determined by the engine parameters $\omega_{H}$ and $\omega_{C}$ according to the condition $\tau_{\mathrm{ad}.}=\tau_{1}+\tau_{2}$. However, the time allocation for the adiabatic processes can be reduced by relaxing the frictionless requirement. There are many ways to do that. Ideally, one would apply the methods of optimal control theory to ω(t) for each of the adiabatic processes in order to optimize the average power over the whole cycle while satisfying the limit-cycle requirement expressed by Equation (21). However, this calculation would be very complex and it would not be possible to determine the solution of the optimal control problem by employing analytical methods. Therefore, this analysis goes beyond the scope of this work. Instead, here we consider the simplified case for which $\tau_{1}$ and $\tau_{2}$ are reduced by the same factor. As can be seen from Figure 4b this method leads to a maximum improvement of ≈9% within the parameter region shown in the graph. As for Figure 4b, the improvement would be even greater for higher values of $T_{H}/T_{C}$.

## 5. Conclusions

We discussed the finite-time performance optimization of the quantum Otto cycle by considering two different well-known models for the working fluid: an ensemble of harmonic oscillators and an ensemble of spin systems. Moreover, we considered both the power optimization of the engine-cycle and the cooling power optimization of the refrigeration-cycle.

The optimization variables are the time allocations of the four processes composing the thermodynamic cycle. In contrast to the majority of studies within this field, we considered the unconstrained optimization problem. This means that the two adiabatic processes were not frictionless: we allowed for some friction generation in order to reduce the duration of the cycle and improve the average power production.

We used analytical techniques to compute the derivative of the work production for a limit-cycle trajectory with respect to the time allocation for the adiabatic processes. We explicitly show that for a frictionless cycle the derivative is zero: the work is a relative maximum. This result immediately implies that the power cannot be optimal for a frictionless cycle. In particular, the globally optimal point must be searched in the region of the configuration space for which the time allocation for the adiabatic processes is shorter than that of the minimum time frictionless process.

Numerical computations have been used to obtain the performance improvement with respect to the constrained optimal power. Depending on the engine parameters, the improvement can be quite significant. The next logical step would be to formulate this problem as a control problem and find the frequency time-dependence leading to maximum power.
